# The role of (observed) gaze behaviour in identity recognition

**DOI:** 10.3758/s13423-026-02908-5

**Published:** 2026-04-14

**Authors:** Lindsay M. Peterson, Colin W. G. Clifford, Colin J. Palmer

**Affiliations:** 1 School of Psychology, UNSW Sydney, Sydney, NSW 2052 Australia; 2 Department of Psychology, National University of Singapore, Singapore, Singapore

**Keywords:** Face recognition, Gaze perception, Gaze behaviour, Eye-tracking, Biological motion

## Abstract

Recognising familiar people is an important function of the human visual system that supports daily social interactions. In addition to static visual cues (e.g., face shape and texture), biological motion cues such as idiosyncratic facial motion and gait contribute to identity recognition. Surprisingly, recent research has indicated an individuals’ *eye movements* exhibit idiosyncratic patterns that contain identifiable information, though it is largely unknown to what extent human observers exploit this information. The current study measures sensitivity of human observers to idiosyncratic gaze behaviours when identifying familiar faces. In two experiments, participants familiarised themselves with faces that were generated from three-dimensional scans of human heads. As participants examined each face, the face’s eye movements were animated using eye-tracking data from real observers such that the face appeared to be looking around the room. Participants were then tested on how much had been learned about each identity’s gaze behaviours. In Experiment 1 (*N* = 40), we found a small but significant effect of gaze behaviour in spontaneously biasing the perceived facial identity even when other visual cues to facial identity were available. In Experiment 2, participants (*N* = 51) were sensitive at discriminating the identity of a face based purely on its eye movements after explicit instructions to rely on gaze behaviour to distinguish different individuals. Our results demonstrate an individual’s dynamic gaze behaviours can inform how others recognise them, expanding current understanding of the visual cues that contribute to identity recognition and the sensitivity of human observers to others’ attentional behaviour.

## Introduction

Recognising familiar people is a critical component of daily human functioning that facilitates social interactions. The visual system relies on a range of facial cues to recognise a person, including their face shape and skin pigment, internal features such as the nose and mouth, and external features such as hair (Abudarham et al., 2019; Burton et al., 1999; Ellis et al., 1979). Non-face cues also support person recognition. In particular, biological motion – natural patterns of movement generated by humans and animals – is a reliable and dynamic, non-face cue used for person recognition (Yovel & O’Toole, 2016). For example, detecting idiosyncrasies in gait and gestures can contribute to identifying a known person (Loula et al., 2005; O’Toole et al., 2002; Yovel & O’Toole, 2016). The current study investigates whether *gaze behaviour* can influence identity recognition, as a form of biological motion that is intimately linked to our perception of another person’s attentional state.

The way that a person visually examines their environment tends to be salient to us in part because it can be indicative of their current state of mind and future goals (Baron-Cohen, 1997). Much of the gaze-perception literature has focused on the static element of gaze (e.g., perceiving or following the direction of a person’s gaze), but the temporal dynamics of gaze may be just as important to us as observers for understanding another person’s mental state. The temporal order of fixations carries information about the gazer’s environment: watching a social interaction through the ‘eyes’ of another whose fixations have been temporally scrambled significantly impacts the interpretation of the interaction (Bush et al., 2015). Saccades (rapid eye movements between fixations) occur up to five times per second while viewing an image (Martin et al., 2018; Wolfe, 2007), and these dynamic behaviours regulate the conversation flow in dyadic interactions (Degutyte & Astell, 2021). Indeed, human observers have a sense of the dynamic gaze behaviours that are socially appropriate given the context (Landmann et al., 2024).

There is reason to think that this sensitivity of human observers to the spatiotemporal dynamics of other people’s gaze behaviour might also extend to the discrimination of the gaze behaviours of different individuals. In neurotypical observers, there is evidence of significant individual differences in gaze behaviour that are stable across time (Berlijn et al., 2022; de Haas et al., 2019; Guy et al., 2019; Mehoudar et al., 2014) and partially heritable, such that identical twins tend to share a greater similarity in how they free-view naturalistic images when compared to dizygotic twins or other individuals (Constantino et al., 2017; Kennedy et al., 2017). Eye movements carry sufficient identifiable information that computer algorithms can be developed to identify an individual from their gaze patterns (termed ‘gaze fingerprinting’; Crockford et al., 2023), but can *human observers* recognise the dynamic gaze behaviours of different individuals? Ziman et al. (2023) reported that observers could learn to correctly distinguish two people from their eye movement data with ~ 60% accuracy when eye movements were represented as a dynamic marker on an image. Earlier studies using a similar methodological approach differed somewhat in their findings: while observers could discriminate their own gaze patterns from random or computer-generated gaze patterns, they found it difficult to discriminate their own gaze patterns from another person’s (Clarke et al., 2017; Foulsham & Kingstone, 2013; Võ et al., 2016). Importantly, however, these past studies did not assess the role of eye-movement behaviours in *face recognition*, but rather how participants can learn to identify patterns in eye-movement data when presented in an abstract manner (e.g., as a moving dot superimposed on an image). In daily interactions, the input to our visual system associated with (observed) gaze behaviours are the biological motion cues produced directly by the movement of our partner’s eyes and face. Are these dynamic visual cues associated with gaze behaviour a component of how we perceive facial identity?

The aim of the current study was to measure the influence of (observed) gaze behaviour on the perception of facial identity. We presented participants with highly realistic computer-generated faces that could move their eyes as if they were looking around the room. The eyes of the faces were animated using eye-tracking data recorded from real human observers such that the eye movements of one (computer-generated) face could be controlled using the eye-movement data recorded from one specific (real) person while the eye movements of other (computer-generated) faces were controlled using the eye-movement data recorded from different (real) people. In Experiment 1, we measured sensitivity at discriminating the identity of two people when participants are not explicitly instructed to rely on eye movements to make their judgements and when other established cues to face identity are also available (e.g., face shape and texture). In the learning phase of the experiment, participants saw animations of faces performing eye movements, as if they were looking around the room, and judged the identity of the face and how closely the face was paying attention to them. In the subsequent test phase, participants viewed blends of the two original faces (facial morphs) that varied along a continuum of relative identity strength. Each morphed face performed gaze behaviours associated with both identities presented during the learning phase. Participants again judged the identity of the face and how closely the face was paying attention to them. We hypothesised that if participants implicitly learn about gaze behaviours associated with a particular identity, then this learning would be evident in facial identity judgements in the testing phase. For example, we anticipated that when the facial identity was ambiguous (i.e., a 50/50 morph of the two original faces), perceived identity would be biased towards the identity corresponding to the eye movements. The specific eye movements performed by a face identity during the learning phase were always different from those presented during the test phase, despite being recorded from the same human observer (across different trials of a free-viewing task). Hence, the task was designed to test recognition of idiosyncrasies in a person’s style of looking behaviour, rather than merely testing recognition of a previously seen pattern of eye movements.

## Experiment 1

### Method

#### Participants

Forty participants (mean age = 18.3 years, min. = 17 years, max. 22 years; 32 females, eight males) completed the experiment. An additional two participants completed the experiment, but their data were not recorded due to a technical error. Participants were undergraduate students enrolled in a first-year psychology course at the University of New South Wales (UNSW) Sydney and had self-reported normal or corrected-to-normal vision. No further demographic information was collected from participants, and no screening surveys were used in the experiment. Participants gave informed consent prior to beginning the experiment. The recruitment and experimental procedures were approved by the Human Research Ethics Committee, School of Psychology, UNSW Sydney. A post hoc sensitivity analysis indicated that the experiment had 95% power to detect a medium effect (Cohen’s *d* ≈.6) with α =.05.

#### Stimuli and apparatus

##### Three-dimensional (3D) face models

**Fig. 1:**
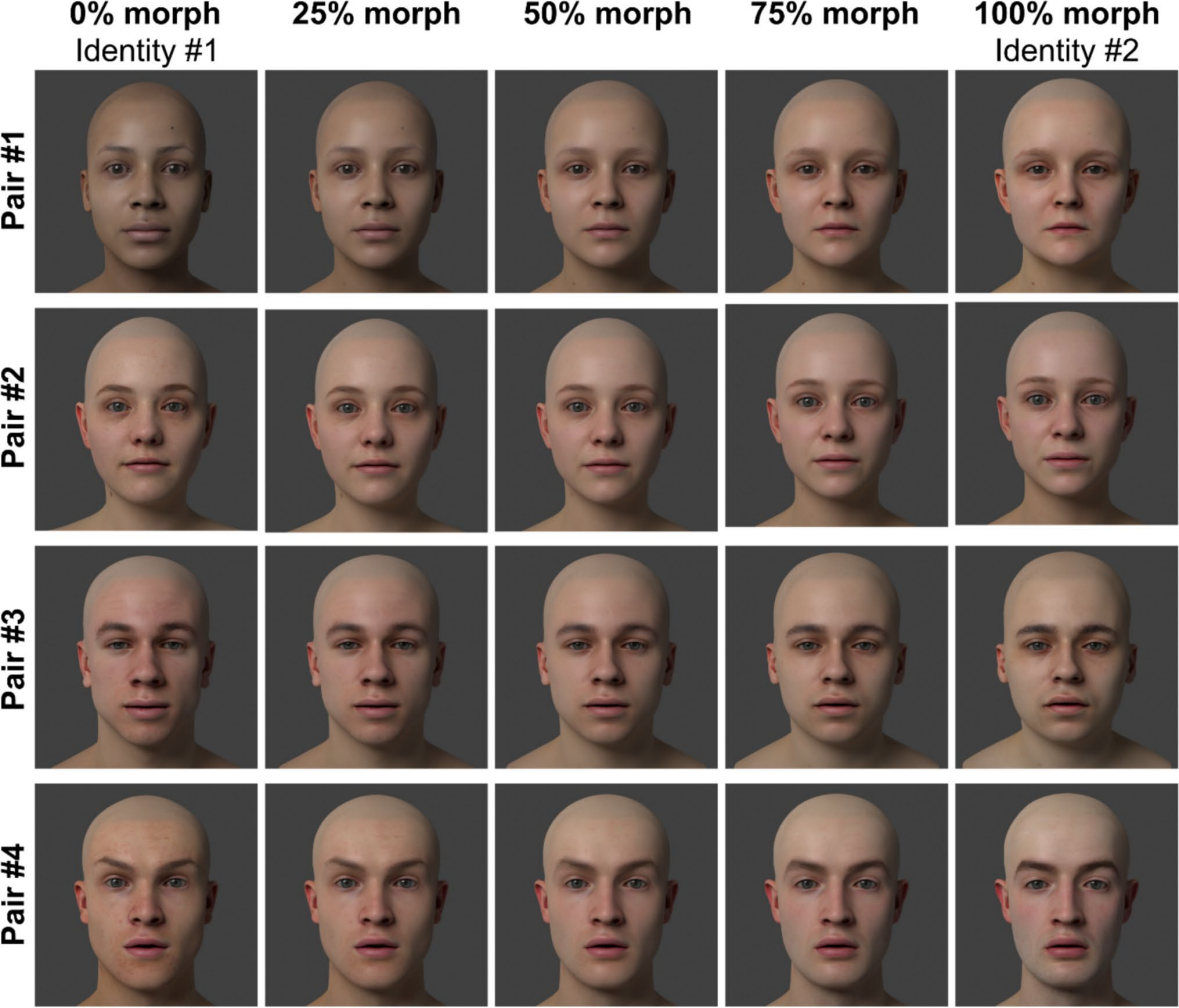
Examples of the stimuli from Experiment 1. There were four identity pairs: two female pairs (#1 and #2) and two male pairs (#3 and #4). Participants were randomly assigned to an identity pair, with equal numbers of participants seeing each pair. Each (unmorphed) face identity can be seen in the far left and right columns. We created morphed faces within each face-identity pair, where the morph percentage is relative to the amount of identity #2 present in the face. For example, a 25% morph face for pair #4 indicates that the morphed face is 25% identity 4.2 and 75% identity 4.1

We used three-dimensional (3D) models of human heads from the 3D Scan Store (https://www.3dscanstore.com/) that had been created from high-definition scans of real individuals. These models were imported into the computer graphics rendering program Blender (version 3.6.2) in their ‘real’ size, with the models having an inter-pupillary distance of approximately 6.3 cm (which corresponds to the average inter-pupillary distance reported in large samples; Dodgson, 2004; Fesharaki et al., 2012). We had eight models in total: two female pairs (referred to as pair #1 and #2) and two male pairs (pair #3 and #4). We refer to identity #1 in pair #1 as identity 1.1, and identity #2 in pair #1 as identity 1.2, and so on. Each face identity can be seen in the far left and right columns of Fig. 1. The 3D models shared the same topology, meaning that the mesh of a 3D model, which defines its 3D shape, could be merged with another face mesh in Blender to create faces that were morphs of two identities. The textures of the two models were morphed in MATLAB and imported into Blender to apply to the morphed 3D mesh. We created morphs ranging from 10 to 90% in 5% increments. The morph percentages are relative to the amount of the second identity present in the morphed face. For example, a 25% morph of pair #2 is a morphed face comprised of 75% identity 2.1 and 25% of identity 2.2. Examples of the morphed faces for each identity pair are shown in the middle three columns of Fig. 1.

##### Animating gaze behaviour

The eye movements of the faces were controlled by the eye-tracking data from the Object and Semantic Images and Eye-tracking (OSIE) dataset (Xu et al., 2014). The OSIE dataset contains the recorded eye movements of 15 human observers that freely viewed 700 images of everyday objects and scenes. The eye-tracking data collected by Xu et al. (2014) were processed to define each participant’s scanpath for each image as a set of fixations (x–y coordinates corresponding to the participant’s point of fixation in an image). Note that the velocity threshold for a saccade to be detected was 22°/s and any unstable fixations (i.e., < 100 ms in duration) were discarded to reduce noise in the eye-tracking data (Xu et al., 2014). As such, the eye movements of the faces in this study consist of a set of fixations but do not include finer-scale eye movements or microsaccades.

We selected four pairs of observers from the OSIE dataset to animate the eyes of the face models in Fig. 1. To maximise the differences in gaze behaviour within a pair, we used an entropy analysis to determine the four most dissimilar pairs of observers in the OSIE dataset based on their scanpaths. For each image in the OSIE dataset, we calculated the dissimilarity between each pair of observers where the x–y coordinates of the fixations in each scanpath were binned, with a total of 192 bins at 50 pixels square for the 800 × 600 pixel image. The durations of each fixation within a bin were made proportional to the total duration of the scanpath, and the distribution of duration-weighted fixations was used to calculate the entropy. Mutual information, calculated as the difference between the conditional entropy (i.e., the average entropy of the two scanpaths) and total entropy for a pair of scanpaths (i.e., the entropy derived from the two scanpaths combined) provided a measure of dissimilarity. Note that mutual information was normalised by the total entropy. By averaging over the dissimilarity for the 700 images we obtained a 15 × 15 observer dissimilarity matrix. We then determined the four most dissimilar pairs by comparing the mean dissimilarity of each observer to every other observer. The selected pairs of observers were assigned to the face pairs; for example, the eye movements of Identity 1.1 (i.e., the face in the top left panel of Fig. 1) were controlled by the observer #7 from the OSIE dataset. Observers #7 and #2 were assigned to pair #1, observers #15 and #5 to pair #2, observers #6 and #4 to pair #3, and observers #12 and #3 to pair #4. We refer to the OSIE observer (eye-movement data) assigned to each facial identity as the *gaze identity.*

**Fig. 2:**
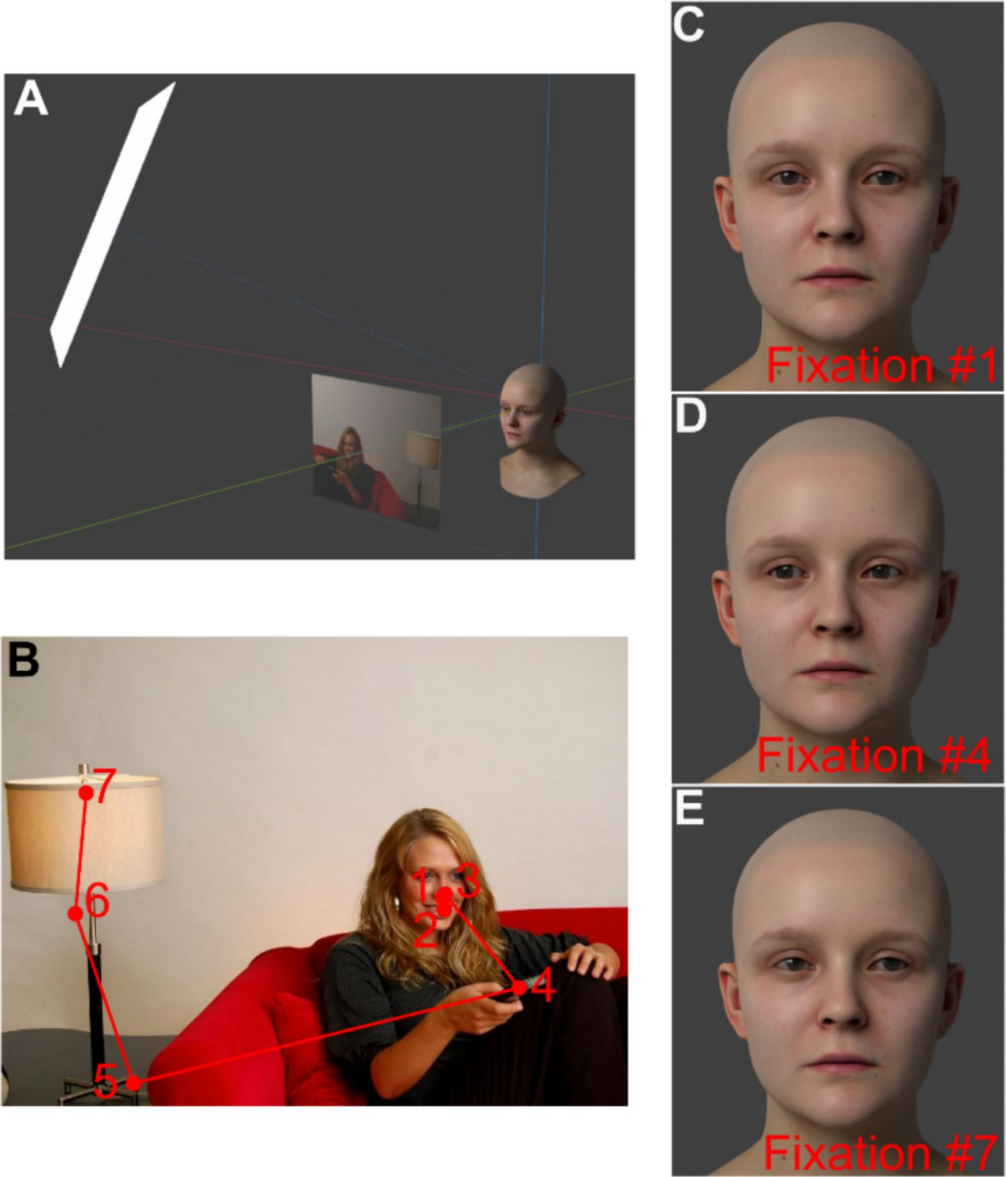
The experimental stimuli were three-dimensional (3D) models of human faces who could look around the room. **(A)** A screenshot of the stimulus setup in Blender. The face fixated on a depth plane 66 cm in front of them and the x–y coordinates of the fixation point were determined by the eye-tracking data from the OSIE dataset. Note that an image from the OSIE dataset has been added to the depth plane such that the plane is visible in this figure; the images were not visible in the experimental stimuli. **(B)** An example of the eye-tracking data from the OSIE dataset. The red dots show each fixation point from this particular scanpath, with the numbers next to each dot conveying the temporal order of the fixations. **(C–E)** The eyes of the face were animated using the eye-tracking data from the OSIE dataset. The eyes in this example are animated with scanpath displayed in **(B).** The first, fourth, and seventh fixations of the scanpath are shown in panels **(C)**, **(D)**, and **(E)**, respectively. The video of the face performing the complete scanpath from **(B)** can be seen in the Online Supplementary Material

We randomly selected scanpaths for 50 images from the OSIE dataset for each identity pair. In Blender, the eyes of each face model were rotated to track the location of a gaze target. This target was located 66 cm in front of the model to match the viewing distance at which the eye-tracking data was recorded in the OSIE dataset (Xu et al., 2014; see Fig. 2A). The position of the target on this depth plane was given by the x–y coordinates from the eye-tracking data (see Figs. 2C–E). We used the fixation durations that were recorded for each fixation in a scanpath such that each image of the model performing a fixation would be displayed for the appropriate duration. Additional fixations were appended to the beginning and end of each scanpath, such that animation always began and ended with a 500-ms central fixation (i.e., the face looking directly ahead). Within a face-identity pair, each face was rendered performing the 50 scanpaths by the gaze identity. This resulted in 100 stimuli for each face-identity pair. As described previously, morphed faces were generated for each face-identity pair. There were 17 morph levels: 10–90% in 5% increments. For each morphed face, eight images were selected from the OSIE dataset and each morphed face was rendered performing the associated scanpaths for each gaze identity within a face pair. This resulted in 272 morphed stimuli in total for each identity pair. Examples of the animated stimuli can be found in the Online Supplementary Material.

In Blender, each model was illuminated by a 60-cm square plane light source that emitted light uniformly across its surface. This light source was positioned 1 m from the model and can be seen on the left in Fig. 2A. The virtual camera was positioned 57 cm in front of the model and had a spatial resolution of 1,280 pixels × 720 pixels. Note that the position of the virtual camera was consistent with participants’ viewing distance during the experiment (~ 60 cm), such that the perspective onto the faces rendered in the images was consistent with the viewing conditions used in the experiment. The rendering engine Cycles was used to render the images in Blender. During the experiment, the stimuli were presented in colour on linearized Display +  + monitors (Cambridge Research Systems) with a refresh rate of 120 Hz.

#### Design and procedure

Participants completed the experiment in a darkened booth, seated approximately 57 cm from the monitor with their head stabilised by a chin rest. At this viewing distance, the faces were approximately ‘life-sized’ with on-screen inter-pupillary distance of ~ 6.3 cm (Dodgson, 2004; Fesharaki et al., 2012). This viewing distance also corresponds to the interpersonal distance that is preferred for an acquaintance or close friend (Sorokowska et al., 2017). Before beginning the task, participants were instructed that they would be learning to recognise two people: Sam and Charlie. These names were selected as these are gender-neutral names in the Australian context in which the study was conducted (i.e., Sam is a common nickname for both the masculine Samuel and feminine Samantha; Charlie is a nickname for the masculine Charles or feminine Charlotte). The instructions explained that on each trial, Sam or Charlie would appear on the screen and move their eyes as if they were looking around the testing booth. Participants were instructed to indicate whether the person on-screen was Sam or Charlie and rated how closely the person was paying attention to them on a 7-point Likert scale (‘Not at all’ to ‘Very closely’). The purpose of this rating response was to prompt participants to pay attention to the eye movements of the face without explicitly stating that this could be a cue useful for identity recognition.

The first or learning phase of the experiment consisted of 100 trials, with 50 scanpaths performed by each facial identity. The faces presented in this phase were always the original, non-morphed faces (i.e., 0% morph and 100% morph, see far left and right column of Fig. 1) with each face consistently paired with the same gaze identity. Participants were randomly assigned to a face/gaze identity pair, and the assignment of the names ‘Sam’ and ‘Charlie’ to each identity was randomised across participants. The presentation order of the stimuli was also randomised for each participant, and participants had the opportunity to take a rest break halfway through the learning phase. Participants received feedback on the correctness of their response via ‘Correct’ or ‘Incorrect’ appearing on the screen after their response was recorded.

The second or testing phase of the experiment consisted of 288 trials: 136 scanpaths performed by each gaze identity plus 16 catch trials. For the catch trials, participants viewed unmorphed faces with the congruent gaze identity, with eight trials each for Sam and Charlie. The scanpaths used to animate the faces’ eye movements in the test phase had not been seen in the learning phase. In other words, these were scanpaths recorded from the same two (human) observers as those used in the learning phase, but when looking at different images in the OSIE dataset. Participants indicated whether the face looked more like Sam or Charlie and did not receive feedback on the correctness of their response. Participants also rated how closely they felt the face was paying attention to them. Participants could take a break halfway through the testing phase and were debriefed by the experimenter upon completing the experiment.

#### Analysis

The experiment produced two sets of data for each participant: data from the learning phase and the testing phase. The identity discrimination responses from the learning phase were summarised as proportion of correct responses and, as this was not a particularly difficult task, we anticipated that participants would perform quite well. We did not plan to conduct any statistical analyses on these data though the descriptive statistics served as a way to check whether participants were paying attention during the experiment (and not randomly pressing buttons, for example). Likewise for the rating data from the learning phase, we did not have specific hypotheses for the ratings of how closely the face was paying attention to the participant, and this question was included in the task to prompt participants to pay attention to the eye movements of the face.

For the testing phase, there were eight data points for each facial morph level for each gaze identity. These data points were converted to proportion of trials in which a participant indicated that the face looked like identity #2, where identity #2 could be Sam or Charlie depending on the random name assignment. A Logistic curve was fit to the data with parameters for the point of subjective equality (PSE), the slope of the function, and the lapse rate. Our hypothesis centred on the PSE: the point on the morphed identity continuum at which the fitted proportion of responses of the two identities was equal. If gaze behaviour does not influence identity recognition, we would expect no difference between the PSEs associated with each gaze identity. For example, if the PSEs for both functions (i.e., the responses for the morphs paired with each gaze identity) are 50%, this indicates that when 50% of face identity #2 is present in the morphed face, participants are equally likely to judge the face as Sam or Charlie. However, if gaze behaviour does influence identity recognition, the PSEs for each gaze identity would be significantly different from one another. Keeping with the example, for morphed faces paired with gaze identity #1, we would expect the PSE to be greater than 50% as the perceived identity is biased towards the gaze identity and therefore, a greater percentage of face identity #2 is required for participants to be equally likely to judge that face as Sam or Charlie. Conversely, for morphed faces paired with gaze identity #2, the PSE would be less than 50% because participants are biased by the gaze identity and a smaller percentage of face identity #2 is necessary in the morphed face for participants to be judging the facial identity at chance.

### Results

**Fig. 3:**
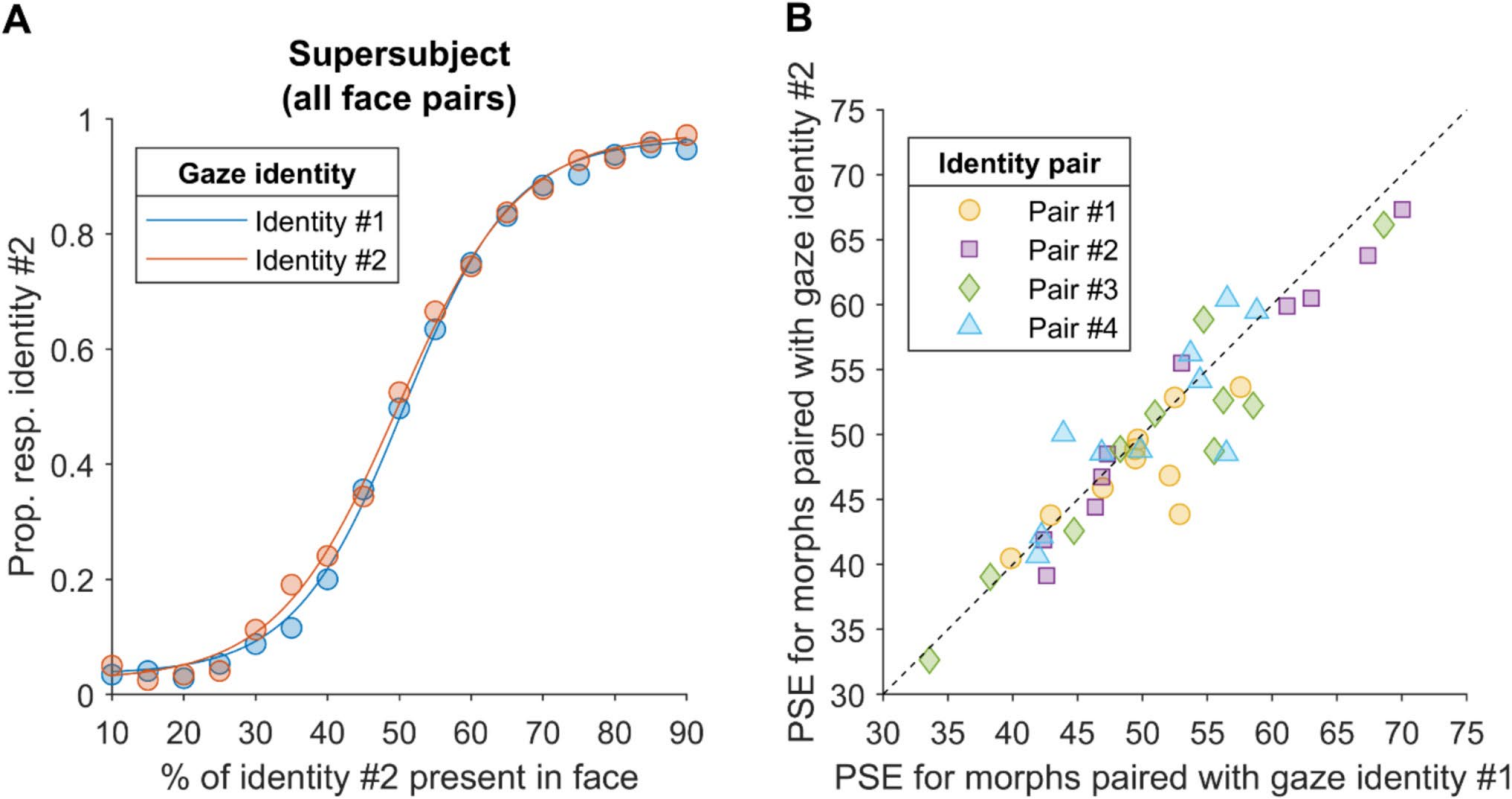
The results from Experiment 1. **(A)** The supersubject data from the testing phase. The percentage of facial identity #2 present in the morphed face is on horizontal axis and the proportion of trials in which the face was reported to be identity #2 is on the vertical axis. The blue circles represent the observed data for trials where the morphed face’s eye movement were controlled by gaze identity #1, and the orange circles correspond to trial where the face’s gaze was controlled by gaze identity #2. The blue and orange solid lines represent the best fitting Logistic function. **(B)** The points of subjective equality (PSEs) for each gaze identity, with the circle markers depicting the PSEs for participants assigned to identity pair #1, square markers depicting identity pair #2, diamond markers depicting identity pair #3, and triangle markers depicting identity pair #4. If gaze behaviour biased the perceived facial identity, then the PSEs for gaze identity #1 should be relatively greater than the PSEs for gaze identity #2 and fall below the dashed line. The fitted data for each participant can be found in the Online Supplementary Material. A plot showing the relationship between each participant’s PSE difference (i.e., PSE for identity #1—PSE for identity #2) with the difference in the Likert ratings for each learned identity (i.e., mean rating for identity #1—mean rating for identity #2) can also be found in the Online Supplementary Material

All participants had accuracy equal to or greater than 85% for the identity discrimination in the learning phase and the catch trials in the testing phase, indicating that participants successfully learnt the names associated with each face. The supersubject data for face identity judgements in the testing phase are depicted in Fig. 3A. As anticipated, participants were more likely to judge that a face was face identity #2 when a greater proportion of identity #2 was present in the morphed face (i.e., in terms of shape and texture cues). In contrast, there was a much weaker effect of gaze behaviour on facial identity judgements. Each participant’s PSEs are plotted in Fig. 3B, contrasting facial identity judgements when the same facial morphs performed gaze behaviours consistent with either gaze identity #1 or gaze identity #2. If participants were biased by gaze behaviour when judging facial identity, the PSEs for gaze identity #1 should be relatively greater than the PSEs for gaze identity #2 and fall below the dashed line in Fig. 3B. Averaged across participants, the PSE for gaze identity #1 (*M* = 51.19, *SEM* = 1.30) was slightly greater than the PSE for identity #2 (*M* = 50.10, *SEM* = 1.24). The difference between the PSE for each gaze identity (across face pairs) was significantly greater than zero (*t*(39) = 2.17, *p* =.036, 95% CI [0.07, 2.10], Cohen’s *d* = 0.34).

However, the bootstrapped 95% confidence intervals for the PSE difference for the supersubject data indicate that the difference was not significantly different from zero (95% CI [−0.02, 1.81]). We also calculated the bootstrapped 95% confidence intervals around the PSE difference for each identity pair (i.e., those shown in Fig. 1) and found that the difference was significantly greater than zero for pair #3 (95% CI [0.23, 3.77]) but not for the other three pairs (pair #1: 95% CI [−0.23, 3.38]; pair #2: 95% CI [−0.89, 2.67]; pair #4: 95% CI [−2.71, 1.41]). We note that the *t*-test with the individual participant data takes the between-subject variability (i.e., the noise along the line of unity in Fig. 3B) into account, while the supersubject analysis does not. As such, the discrepancy between the results based on the individual participant data and the supersubject data is possibly due to the masking of between-subject variability in the supersubject data rendering this analysis less powerful. Furthermore, the inconsistency in the PSE difference across identity pairs in the supersubject data could reflect variation in the discriminability of facial features or gaze behaviour across pairs. In summary, we find some evidence for a significant effect of identity-specific eye movements on facial recognition, but this effect may only be significant when discriminating between certain facial identities using sufficiently dissimilar gaze behaviours and is clearly very small compared to the influence of static face features on facial identity judgements (i.e., face shape and texture).

## Experiment 2

Experiment 1 produced limited evidence of identity-specific eye movements spontaneously biasing facial recognition. That the effect observed was relatively weak is perhaps unsurprising given that traditional cues to facial identity (e.g., face shape and texture) were available to participants. Furthermore, participants were not explicitly instructed to base their judgements of identity on the face’s gaze behaviour, thus the effect observed represents spontaneous or implicit learning of individual differences in gaze behaviour when observing others. The aim of Experiment 2 was to measure participants’ sensitivity at discriminating the identity of two people based on their gaze behaviour when facial shape and texture could not be relied upon to discriminate identity. Participants were explicitly instructed to scrutinise the eye movements of the two people as they would be tested on how much they learned about the gaze behaviours. Eye-movement data recorded from the same two individuals was later presented via a computer-generated face that did not vary across trials in shape or textural features, and participants were tasked to identify which of the two earlier-presented identities the gaze behaviour of the face was consistent with. We theorised that explicitly prompting participants to focus on gaze behaviour would elicit best possible performance on the identity discrimination task.

### Method

#### Participants

Fifty-one participants (mean age = 19.5 years, min. = 18 years, max. = 25 years; 39 females, 12 males) completed the experiment. The sample size was informed by a power analysis that indicated approximately 50 participants would be required to detect a small effect (Cohen’s *d* ≈ 0.4) with 90% power and α =.05. The recruitment procedure was as described for Experiment 1.

#### Stimuli and apparatus

**Fig. 4:**
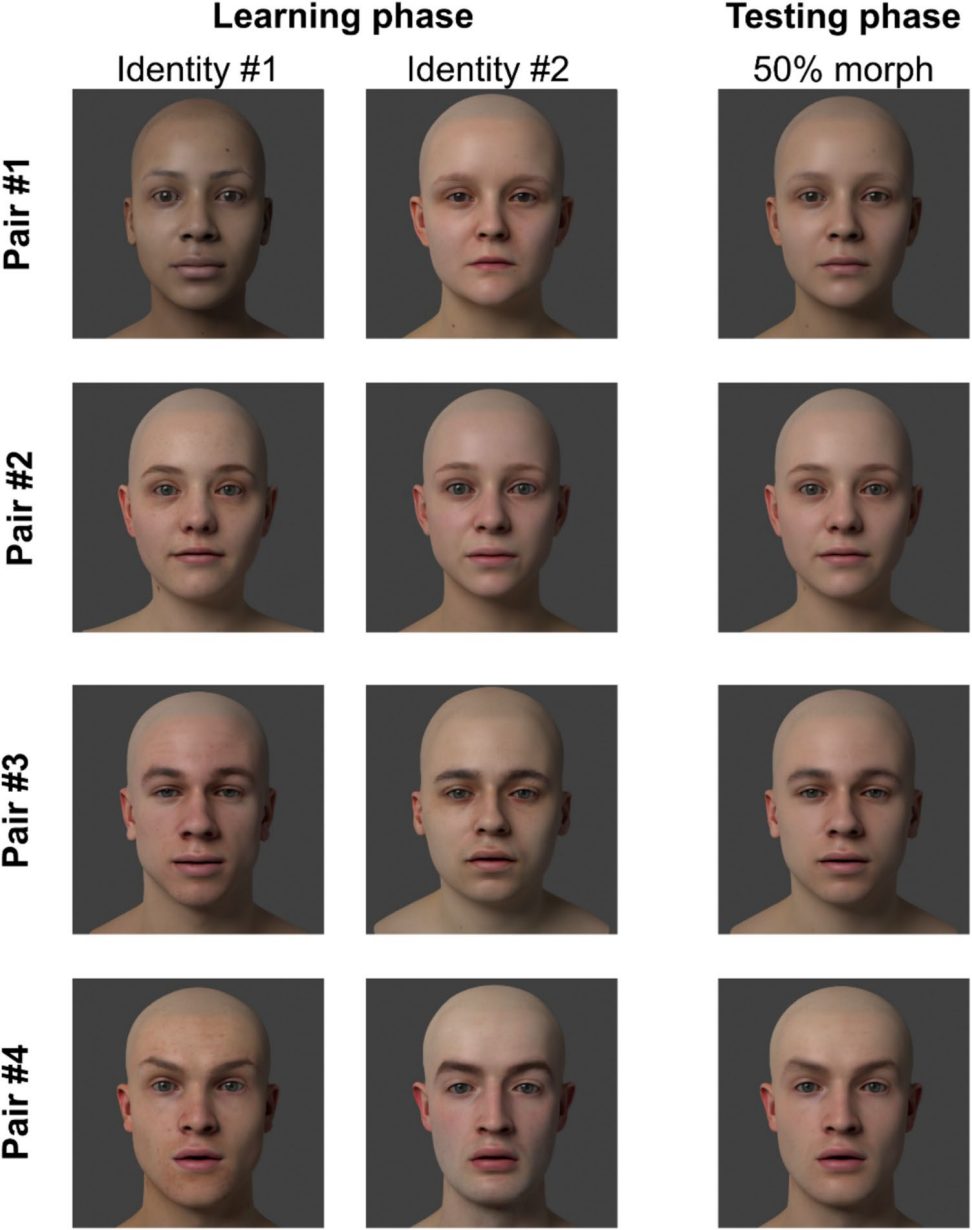
Examples of the stimuli from Experiment 2. There were four identity pairs: two female pairs (#1 and #2) and two male pairs (#3 and #4). Participants only saw one identity pair and identity pairs were randomly assigned to each participant. In the first part of the experiment, participants learned to recognise each identity. On each trial, one identity would appear and appear to look around the screen. In the second part of the experiment, participants would see the 50% morph of the two faces (shown in the right column) and judge whether the face looked around in a manner that was more similar to identity #1 or identity #2

The same 3D head models from Experiment 1 were used to create the stimuli, and the OSIE dataset was again used to animate the eyes of each face. The pairing of facial identities remained the same, as well as the gaze identity associated with each face. The key difference in Experiment 2 was that only 50% morph faces were created, rather than the larger range of 10–90% facial morph used in Experiment 1. In particular, all faces presented in the test phase of the experiment were 50% morphs so that participants were now only able to base their responses on changes in the face’s eye movements across trials, rather than changes in static face features (e.g., face shape and texture). Examples of the stimuli from Experiment 2 can be seen in Fig. 4. Similar to Experiment 1, 50 scanpaths were randomly selected from the OSIE dataset for the learning phase and each unmorphed face rendered performing the scanpath from the associated gaze identity. Another 100 images were randomly selected from the OSIE dataset and the associated scanpaths were used to animate the gaze of each morphed face. Within a pair, a scanpath was not repeated across the morphed and non-morphed faces such that a scanpath performed by a particular individual was never seen twice by a participant. The morphed face performed the scanpath from the gaze identities assigned to the facial identity pair. This resulted in 200 stimuli for each morphed face.

#### Design and procedure

The experiment had two phases: the learning phase and the testing phase. In the learning phase, participants were introduced to the two identities (Sam and Charlie) and instructed that they would be learning to recognise them. On each trial, Sam or Charlie would appear on-screen and their eyes would move as if they were looking around the room. Participants were instructed to indicate whether they saw Sam or Charlie on each trial, and to pay close attention to the way Sam or Charlie looked around, as they would be tested on how much they had learnt about Sam and Charlie’s looking behaviours in the second part of the experiment.

The learning phase consisted of 100 trials in total, with 50 trials for each facial identity. The name assigned to each identity (i.e., Sam or Charlie) was randomised for each participant. On each trial, the identity appeared on the screen and performed the gaze behaviour. A response screen then appeared with the response prompt: “Did you see Sam or Charlie? Press the left arrow key for Sam or the right arrow key for Charlie.” Participants received feedback on the correctness of their response with ‘Correct’ or ‘Incorrect’ appearing on the screen following their response. The order of the trials was randomised, and participants were given the opportunity to take a break halfway through the learning phase.

Following the completion of the learning phase, participants were informed that they would now be tested on how much they learnt about Sam and Charlie’s gaze behaviour. In the testing phase, participants would see a new facial identity (which was a 50% morph of the two face identities shown in the learning phase), but their eye movements would still be controlled by either Sam or Charlie. Participants indicated whether this new person looked around the room in a way that was more like Sam or Charlie. There were 200 trials in total, with half of the gaze behaviours performed by Sam and half by Charlie, using scanpath exemplars for these identities that had not been seen in the learning phase (i.e., scanpaths from the OSIE dataset that were recorded from the same human observers when looking at different images). Hence, accurate performance in the test phase relied on participants learning differences in the style of looking behaviour displayed by the two identities during the learning phase, rather than simply recognising specific scanpaths that they had seen before. The order of trials was randomised for each participant, and there was a rest break at halfway through the testing phase.

Upon completing the experiment, a small text box appeared on-screen that prompted participants to respond to the question: “How did you decide whether the person's eye movements were more like Sam's or Charlie's?” Once the participant had entered their response, the experiment was finished and the participant was debriefed by the experimenter.

#### Analysis

The data from the testing phase were analysed using signal detection theory. We calculated the proportion of identity #1 responses on the trials where the gaze behaviour was performed by identity #1 (‘hits’) and on trials where the gaze behaviour was performed by identity #2 (‘false alarms’). This allows for the separation of discrimination sensitivity from participants’ overall tendency to report that a particular person (i.e., identity #1 or #2) was performing the looking behaviour (i.e., their decision criterion or response bias). Discrimination sensitivity (*d’*) was given by:

$${d}{\prime}=z({P}_{H})-z({P}_{FA})$$

where the z-score for the false alarm rate is subtracted from the z-score for the hit rate (Kingdom & Prins, 2010). Note that the maximum possible discrimination sensitivity value was 5.15. The decision criterion (*C*) was calculated from:

$$C= -0.5(z({P}_{H})+z({P}_{FA}))$$

where an unbiased participant would have a criterion of zero, and negative values correspond to a participant having a bias towards judging the gaze behaviour as being performed by identity #1 on a given trial (Stanislaw & Todorov, 1999).

### Results

**Fig. 5:**
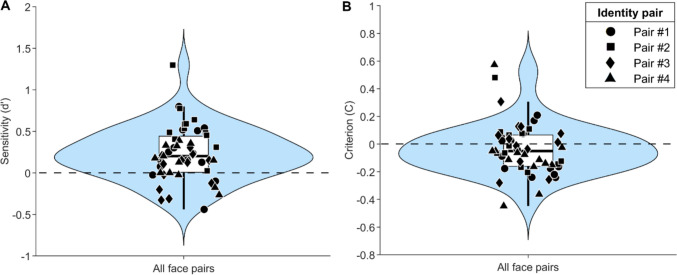
The results from Experiment 2. Sensitivity at discriminating gaze identity is shown in **(A)** and response bias is shown in **(B)**. The black markers represent the data from each participant (the circle markers depict participants assigned to identity pair #1, square markers depict identity pair #2, diamond markers depict identity pair #3, and triangle markers depict identity pair #4), the horizontal black bar is the median, the lower and upper boundaries of the white box are the 25th percentile and 75th percentile, and the black lines extending from the box represent the range of the data, excluding outliers. The probability density estimate of the data is shown in blue

Discrimination sensitivity and criterion for each participant is shown in Fig. 5. Overall, accuracy (*M* = 54.83%, *SEM* = 0.86%) and sensitivity were quite poor (*M* = 0.23, *SEM* = 0.04), indicating that the task was generally a difficult one, but there is evidence that some participants could successfully discriminate idiosyncrasies in looking behaviour. Discrimination sensitivity was significantly greater than zero (*t*(50) = 5.03, *p* <.001, Cohen’s *d* = 0.70), a large effect that indicates that participants were able to discriminate identity based on looking behaviour to some extent. We note that there is some variation in the mean sensitivity values across identity pairs: pair #1: 0.22, pair #2: 0.55, pair #3: 0.02, pair #4: 0.12. We also note the range of *d’* values depicted in Fig. 5A: the minimum *d’* is −0.44 and the maximum *d’* is 1.30, with approximately 20% of participants having a *d’* greater than 0.5 and 75% of participants with a *d’* greater than zero. Taken together, our results indicate that participants can learn to recognise an individual’s pattern of looking behaviour, even when that looking behaviour is derived simply from eye movements recorded during the free viewing of naturalistic images. As the eye movements during the learning and test phases were always different (though recorded from the same two real observers while looking at different naturalistic images in a free-viewing task), these results reveal an ability of our participants to learn ‘styles’ of looking behaviour when viewing another person’s face rather than simply recognising previously seen animations. The criterion was not significantly different from zero for any of the identity pairs (Fig. 5B), suggesting that participants were not biased towards selecting one identity over another (pair #1: *t*(13) = −1.39, *p* =.187; pair #2: *t*(11) = 0.06, *p* =.958; pair #3: *t*(11) = 0.06, *p* =.952; pair #4: *t*(12) = −1.40, *p* =.188).

### Discussion

Facial recognition is supported by a number of visual cues, including holistic facial structure, skin pigmentation, and internal and external features of the face (Abudarham et al., 2019; Burton et al., 1999; Ellis et al., 1979). However, successful identity recognition often goes beyond these traditional visual cues and involves non-face cues as well as personal knowledge of the individual and the emotional response associated with the individual (Gobbini & Haxby, 2007; Gobbini et al., 2004). In general, dynamic cues, such as facial motion, gestures, and gait are known to support recognition (Loula et al., 2005; O’Toole et al., 2002) though the extent to which gaze as a form of biological motion influences recognition and informs the internal representation of an individual is unclear. The current study contributes to this literature by demonstrating that human observers can learn about an individual’s idiosyncratic gaze behaviours, both spontaneously and when explicitly instructed to do so, and this learning can influence identity recognition (though this effect was only found for some identities in Experiment 1). These results lay a foundation for future studies to examine further how the dynamics of (observed) gaze behaviours inform person recognition and other aspects of person perception.

The current results are consistent with eye-tracking studies that have highlighted that individual differences in gaze behaviour are stable over time (Berlijn et al., 2022; de Haas et al., 2019; Guy et al., 2019; Mehoudar et al., 2014). These differences in eye movements across individuals are not simply random variation but rather idiosyncratic gaze behaviours carrying identifiable information about an individual, such that computer algorithms can be developed to identify individuals from their eye-movement data (Crockford et al., 2023). Few studies have examined gaze-based identity recognition (in human observers), and these studies suggest that discrimination of identity from eye-movement data is possible but limited (Clarke et al., 2017; Foulsham & Kingstone, 2013; Võ et al., 2016; Ziman et al., 2023). However, the aforementioned studies used an abstract stimulus in which eye movements were represented by a dynamic dot on an image. In contrast, this study demonstrates that eye movements can contribute to identity recognition when observed naturalistically as patterns of facial motion, even when more traditional and robust cues to facial identity are present.

Participants reported that different aspects of each face’s gaze behaviour influenced their responding, including the number of fixations, fixation duration, and spatial distribution of the fixations. Some participants also associated an emotional state with each identity. For example, participants described associating slower and subtler eye movements with a particular person, and this person was perceived as being calm and relaxed. Conversely, a person that became associated with more rapid eye movements was described as anxious, nervous, and erratic. These results suggest an interesting avenue for future research: If we can recognise individual patterns of gaze behaviour, how does this influence social judgements made about the individual? Previous research has identified a link between gaze and an individual’s emotional state (e.g., Mathews et al., 2003) though much of this research has used static stimuli that do not convey the dynamic nature of gaze. Recent research from Landmann et al. (2024) revealed that affective evaluations are made about specific gaze behaviours. For example, observers tended to rate fast blinking and gaze shifts more negatively than slower eye movements, with the former behaviours associated with restlessness and inattentiveness. The evaluations of gaze were modulated by the emotion context: for example, downwards gaze was deemed more socially appropriate during an emotionally negative conversation (Landmann et al., 2024). Together with the current study, these results suggest that future research focusing on social evaluations of an individual’s dynamic gaze behaviour may deepen understanding of gaze perception and its role in social cognition.

Another potential avenue for future research is to test whether providing context for the target of the face’s gaze enhances the influence of gaze behaviour on the perceived facial identity. In the current study, participants were informed that the face was moving their eyes as if they were looking around the room. In reality, the eye movements were driven by eye-tracking data recorded from human observers as they free viewed a set of naturalistic images. As such, any learning about the face’s gaze patterns is likely due more to the mechanics of the eye movement, such as number of fixations or the dispersion of the fixations, rather than the alignment of the fixation pattern to the subject’s environment. The stimuli used in the current study could be adapted, however, to include an image being examined by the face, similar to the stimuli used by Pantelis and Kennedy (2017), which consisted of a human face behind a semi-transparent screen that displayed images. The participant could see both the face and its eye movements and the image being examined by the face. This type of stimulus could be used to examine whether individual differences in the types of visual features that attract attention (e.g., low-level vs. semantic-level features of the visual environment; Wang et al., 2015) are salient to observers when viewing others and contribute to person perception. Since Ziman et al. (2023) suggests that an individual’s eye movements can be recognised with visual context, it is possible that the small effects reported in the current study would be bolstered by providing additional information about the gazer’s visual environment. Alternatively, a face’s eye movements could be controlled by eye-tracking data recorded from within the experimental testing room, such that participants are viewing how another person interacts with the shared visual environment.

The current study investigated the extent to which human observers can discriminate the dynamic gaze behaviours of different individuals. We report that eye movements can serve as a motion cue for identity recognition, though discriminating identity based solely on eye movements is challenging. Our research opens up a number of different avenues for future research that will be informative for understanding gaze perception, visual attention, and social cognition. One question is whether the demographics of an observer and those they are interacting with influence how gaze behaviours are perceived. For example, future studies could examine how gender-related biases may affect the results reported in the current study, given that the gender of a face can influence how others perceive both its direction of gaze and facial expression (Slepian et al., 2011). Another avenue for future work is the role of neurotypicality, which was not examined in the current study, but is likely to influence the effects reported here. For example, individuals diagnosed with ASD demonstrate atypical visual attention, often fixating less on semantic image content (such as faces) than neurotypical observers (Amso et al., 2014; Wang et al., 2015) and displaying difficulty identifying the target of another’s gaze (Pantelis & Kennedy, 2017). This raises a question of both how the perception and interpretation of dynamic gaze behaviour differs across individuals and groups, and how differences between people in their gaze behaviour are perceived by others.

## Data Availability

The data and research materials are available by contacting the corresponding author. Access to some research materials may require purchasing third-party software assets. Supplementary material can be accessed via the Open Science Framework at: https://osf.io/fu8yp

## Code availability

The code is available by contacting the corresponding author.

## Declarations

### Open practices statement

The study was not preregistered. The data and research materials are available by contacting the corresponding author. Access to some research materials may require purchasing third-party software assets.
